# Antiglycation Activity of Iridoids and Their Food Sources

**DOI:** 10.1155/2014/276950

**Published:** 2014-12-29

**Authors:** Brett J. West, Akemi Uwaya, Fumiyuki Isami, Shixin Deng, Sanae Nakajima, C. Jarakae Jensen

**Affiliations:** ^1^Research and Development, Morinda, Inc., 737 East 1180 South, American Fork, UT 84003, USA; ^2^Research and Development, Morinda, Inc., 3-2-2 Nishishinjuku, Shinjuku-ku, Tokyo 160-0023, Japan; ^3^Department of Language and Literature, Kyoritsu Women's Junior College, 2-2-1 Hitotsubashi, Chiyoda-ku, Tokyo 101-8437, Japan

## Abstract

Iridoids are dietary phytochemicals that may have the ability to inhibit the formation of advanced glycation end products (AGEs). Three studies were conducted to investigate this anti-AGE potential. First, the inhibition of fluorescence intensity by food-derived iridoids, after 4 days of incubation with bovine serum albumin, glucose, and fructose, was used to evaluate *in vitro* antiglycation activity. Next, an 8-week open-label pilot study used the AGE Reader to measure changes in the skin autofluorescence of 34 overweight adults who consumed daily a beverage containing food sources of iridoids. Finally, a cross-sectional population study with 3913 people analyzed the relationship between daily iridoid intake and AGE accumulation, as measured by skin autofluorescence with the TruAge scanner. In the *in vitro* test, deacetylasperulosidic acid and loganic acid both inhibited glycation in a concentration-dependent manner, with respective IC_50_ values of 3.55 and 2.69 mM. In the pilot study, average skin autofluorescence measurements decreased by 0.12 units (*P* < 0.05). The cross-sectional population survey revealed that, for every mg of iridoids consumed, there is a corresponding decline in AGE associated age of 0.017 years (*P* < 0.0001). These results suggest that consumption of dietary sources of iridoids may be a useful antiaging strategy.

## 1. Introduction

Epidemiological evidence suggests that diets rich in fruits and vegetables may reduce the risk for developing chronic health conditions [1–3]. Fruits and vegetables are dietary sources of nutrients. They are also sources of other biologically active compounds that may promote health, often referred to as phytochemicals. These may have a wide spectrum of possible bioactivities [4]. There are several categories of phytochemicals available in plant based foods. Among these are the monoterpenes, of which iridoids are a subcategory [5]. Iridoids are known to possess a wide range of bioactivities [6]. Among the reported possible health benefits of iridoids is the ability to reduce advanced glycation end products* in vivo* [7, 8].

Advanced glycation end products (AGEs) are a group of compounds that result from a series of nonenzymatic reactions between reducing sugars and protein amino groups [9]. AGEs may be formed in food during cooking with dry heat, such as Maillard browning [10]. But they may also form within the human body as a result of elevated blood glucose [11]. The initial glycation reaction forms a Schiff base, which rearranges to an Amadori product [12]. Amadori products undergo further reactions, including degradation and autooxidation, to form reactive dicarbonyls which form AGE cross-links [13, 14]. Conditions of oxidative stress and production of superoxide anion radicals are also thought to initiate and accelerate the formation of AGEs [15, 16]. AGEs may also bind to cell surface receptors, known as RAGE. This initiates a series of intracellular responses that are mediated via nuclear factor-kappa B, inducing inflammation and reactive oxygen generation, as well as additional transcription of RAGE [17]. These conditions contribute to a cycle which leads to increased tissue damage. Increased AGE levels have been linked to several chronic health conditions and contribute to the aging process [18, 19].

Noni (*Morinda citrifolia*), Asian cornelian cherry (*Cornus officinalis*), European cornelian cherry (*Cornus mas*), and olive (*Olea europaea*) have been used for centuries as foods and to improve health [20–23]. Significant antioxidant activity has been demonstrated for each of these food plants [24–28]. Additionally, reduction of AGEs* in vitro* and* in vivo* reported biological activities of noni, cornelian cherries, and olive leaf extract [7, 29, 30]. Each of these plants produces iridoids [31–33]. As some iridoids have demonstrated anti-AGE activity* in vivo*, the current study was carried out to evaluate the potential of major iridoids in these plants to inhibit glycation reactions, one of the pathways involved in the formation of AGEs. Additionally, a human pilot study and cross-sectional survey were conducted to determine whether the observed antiglycation activity is manifested when dietary sources of iridoids are consumed.

## 2. Materials and Methods

### 2.1. Antiglycation Activity Assay

Phosphate buffer (193 mM KH_2_PO_4_, 73.4 mM Na_2_CO_3_, pH 7.2) was purchased from 3M (Saint Paul, MN). Deacetylasperulosidic acid was purchased from Chengdu Biopurify Pharmaceuticals (Chengdu, China). Loganic acid was purchased from ChromaDex (Irvine, California). Aminoguanidine HCl, bovine serum albumin (BSA), fructose, and glucose monohydrate were obtained from Sigma-Aldrich (St. Louis, MO).

To measure antiglycation activity, a previously described Maillard reaction-based method was followed, but with some modification [34]. Aminoguanidine, the positive control, and the iridoids were evaluated at concentrations ranging from 1.25 mM to 5 mM. This was done by incubating the control and samples for four days, at 58 ± 2°C, in 1 mL phosphate buffered BSA solution (10 mg/mL) containing 25 mM fructose, 25 mM glucose, and 0.02% sodium azide. Reference blanks were also prepared. Many AGEs contain fluorophores which are useful for detection [14]. Following incubation, fluorescence intensities of the blanks, positive control, and samples were measured with a microplate reader, at 360 nm excitation and 460 nm emission. Samples were evaluated in triplicate. For each sample, the minimum concentration that inhibited 50% of glycation (IC_50_) was calculated from the results.

### 2.2. Open-Label Pilot Study

#### 2.2.1. Preparation of Iridoid Enriched Beverage

*Morinda citrifolia* (noni) fruit puree was produced by harvesting fruit in French Polynesia, which was allowed to fully ripen. This was followed by mechanical separation of fruit flesh from the seeds in a micromesh screen in a commercial fruit pulper. The puree was then pasteurized in certified fruit processing facility in Mataiea, Tahiti. Olive leaf extract was prepared by extraction of dried* Olea europaea* leaves with ethanol, followed by removal of the solvent and residual water by evaporation. The resulting extract was milled to a powder with a particle size < 0.420 mm and total moisture < 6%. Ripe* Cornus mas* and* Cornus officinalis* fruits were harvested during the autumn season.* C. mas* fruit was harvested in the Anatolia region of Turkey, whereas* C. officinalis* fruit was harvested in the Shaanxi and Hunan provinces of China. The seed pits were removed from the fruit with automatic fruit pitters. Batches of* C. mas* fruit were processed into puree in an auger press micromesh (4 mm) screen fruit pulper. Depitted* C. officinalis* fruits were dried at low heat and then shipped to the US, where the reconstituted juice was extracted by pumping/filtration through a stainless steel mesh screen. All ingredients were blended and pasteurized at a good manufacturing certified fruit processing facility in American Fork, UT, to produce the iridoid enriched beverage (TruAge Max, Morinda, UT, USA) used for the human pilot study.

#### 2.2.2. Patients and Inclusion/Exclusion Criteria

To evaluate the anti-AGE potential of the iridoid enriched beverage in humans, thirty-four overweight adult volunteers completed an open-label pilot study (trial registration ClinicalTrials.gov NCT01597076). Inclusion criteria were as follows: male or female adults age 25 to 60 years; overweight or obese (BMI 23.0 to 39.9 kg/m^2^); impaired fasting glucose (100 to 125 mg/dL); prehypertension or grade 1 hypertension (systolic blood pressure range of 120–159 mm Hg and diastolic blood pressure range of 80–99 mm Hg); ability to comprehend the full nature and purpose of the study; written informed consent; and a willingness to comply with procedures. Exclusion criteria were as follows: prescription medication use for hypertension, high cholesterol, diabetes, heart disease, cancer, liver disease, or AIDS/HIV; intake of dietary supplements within the previous month; pregnancy or breastfeeding; history of alcohol, drug, or medication abuse; any medical conditions or diseases that may affect subject safety or confound study results; smoking more than one pack of cigarettes per day; allergies to any ingredient in the investigational product; and current participation in another study.

#### 2.2.3. Pilot Study Methods

Participants were asked to consume 60–240 mL of the iridoid enriched beverage daily for 8 weeks. Baseline and poststudy skin AGE levels of the participants were measured with the AGE Reader (DiagnOptics Technologies B.V., Groningen, Netherlands). The AGE Reader is a validated noninvasive instrument which measures skin autofluorescence to determine relative AGE levels in the skin [35]. This human study was conducted under the direction of Dr. Samuel Oetoro at the University of Indonesia (Jakarta, Indonesia) with support from Sprim Advanced Life Sciences (San Francisco, CA) and was approved by the Health Research Ethics Committee of the Faculty of Medicine.

### 2.3. Cross-Sectional Population Study

#### 2.3.1. Data Collection

A cross-sectional population study was also conducted to further evaluate the association between iridoid consumption and skin AGE levels. A questionnaire was used to collect demographic data and information on consumption of dietary iridoids, determined from the current daily ingestion rates of beverages with iridoid containing ingredients (namely, noni, cornelian cherries, and/or olive extracts). The survey was conducted at ten locations in Japan during health education and promotion events and was approved by the research ethics committee of Kyoritsu Women's College (Tokyo). Survey locations included Fukuoka, Hiroshima, Kagoshima, Kanazawa, Nagoya, Okinawa, Osaka, Sapporo, Sendai, and Tokyo. Skin AGE measurements (skin autofluorescence) were recorded for 2,790 consumers of iridoids and compared to those of 1,123 individuals who did not consume iridoids (the reference group).

#### 2.3.2. Data Analysis

Previous studies in healthy populations have demonstrated that skin autofluorescence (AGE score) is positively and linearly correlated to age [36, 37]. Regression analysis of the data from these previous cross-sectional surveys has revealed that AGE score increases by 0.024 per year in most people. The linear equation generated from the regression analysis is useful in predicting an AGE score for anyone of a given age and vice versa. In the current study, AGE associated age (ASA) is defined as the age in years that is most typical or expected with a specific AGE score, as determined from the previous cross-sectional studies. We investigated the effect of iridoids on skin AGE levels by calculating the difference between each participant's age, in our current study, and AGE associated age (ASA). These differences were compared among iridoid consumers and the reference group by analysis of variance and Student's *t*-test. Additionally, linear regression analysis was used to evaluate the magnitude of the effect of iridoid intake on skin AGE levels.

#### 2.3.3. Iridoid Analysis

Samples of the beverages identified in the survey were evaluated for iridoid concentration by high performance liquid chromatography, following methods similar to those previously described [32, 38]. Briefly, 1 g of sample was dissolved in 10 mL 1 : 1 (v/v) water : methanol and then filtered through a 0.45 *μ*m PTFE filter. Iridoid reference standards were dissolved in methanol and diluted to produce standard curves. Chromatographic separations were performed on a Waters 2690 separations module coupled with 996 PDA detectors (Waters Corporation, Milford, MA, USA), equipped with a C18 column. A linear gradient of 100% aqueous formic acid (0.1%) for 0–5 min, followed by 70% aqueous formic acid and 30% MeCN for 40 min, was used to elute samples at a flow rate of 0.8 mL/min. The PDA detector was monitored in the range of 210–400 nm. Iridoids were identified in the samples by comparison of retention times and UV absorbance of compounds in the samples with those of the standards.

## 3. Results

### 3.1. Antiglycation Activity Assay

**Figure 1:**
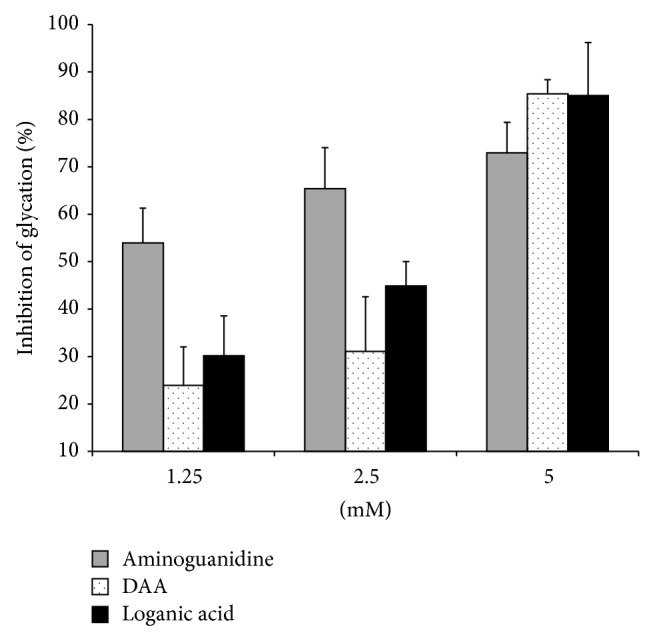
Mean antiglycation activity (with standard deviation) of two iridoids, deacetylasperulosidic acid (DAA) and loganic acid, and aminoguanidine (positive control) at 1.25 mM, 2.5 mM, and 5 mM in the bovine serum albumin/fructose/glucose antiglycation assay. Inhibition of glycation (%) was determined by comparing fluorescence intensities (360 nm excitation, 460 nm emission) of samples and positive control to those of reference blanks.

Deacetylasperulosidic acid and loganic acid inhibited the nonenzymatic glycation of BSA by fructose and glucose (Figure 1). The positive control, aminoguanidine, appears to have greater activity than both iridoids at the lowest two concentrations. In this assay, the aminoguanidine concentration at which 50% of glycation activity was inhibited (IC_50_) was less than 1.25 mM. The mean IC_50_ values (± standard deviation) of deacetylasperulosidic acid and loganic acid were 3.55 ± 0.11 mM and 2.69 ± 0.11 mM, respectively. But at 5 mM, both deacetylasperulosidic acid and loganic acid had greater antiglycation activity than aminoguanidine. In fact, the increase in aminoguanidine activity between 1.25 mM and 5 mM was only 35.3%. Over the same concentration range, deacetylasperulosidic acid activity increases approximately 257%, while loganic acid antiglycation activity increased approximately 182%.

### 3.2. Open-Label Pilot Study

**Figure 2:**
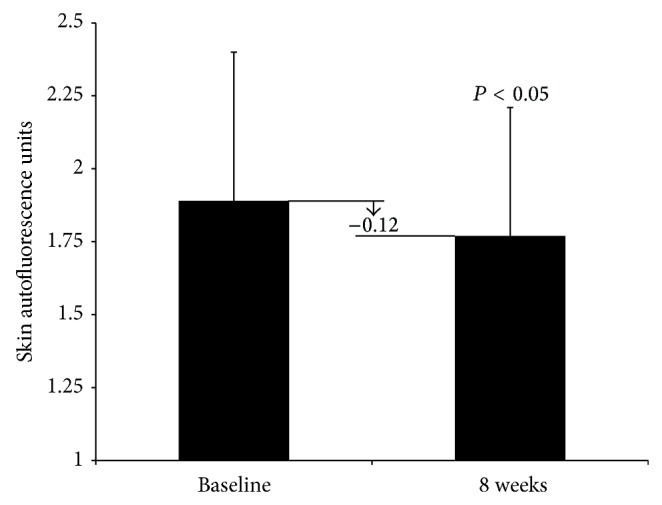
Baseline and week 8 mean skin autofluorescence units (with standard deviation) of 34 overweight adults who consumed daily a beverage containing food sources of iridoids. The average change in autofluorescence was −0.12 units.

In the human pilot study, the average age of the volunteers was 40 years. There were 20 females and 14 males that completed this trial. No treatment related adverse health events were observed. There appeared to be some change in skin AGE levels over the 8-week course of the study (Figure 2). Mean (± standard deviation) baseline skin autofluorescence was 1.89 ± 0.51 units. By week 8, mean skin autofluorescence declined to 1.77 ± 0.44 (*P* < 0.05). During this time, the average change in skin autofluorescence was −0.12 units. The individual change ranged from −1.00 to 0.50. Previously published population reference values [36] indicate that the average baseline value in this study is equivalent to skin autofluorescence values expected in a 44-year-old. Therefore, the average initial AGE associated age (ASA) of those enrolled in the study was 4 years older than their average chronological age of 40. However, after 8 weeks of consuming the iridoid enriched beverage, the average ASA had declined to 39 years.

### 3.3. Cross-Sectional Population Study

Results from the cross-sectional study demonstrate an association between dietary iridoid intake and AGE levels.  Table 1 summarizes the mean differences between participant age and AGE associated age or ASA. The reference group, composed of those who did not consume iridoids, had AGE scores that were not significantly different from those of the general healthy populations previously studied [36, 37]. Those who consumed iridoids, however, had significantly lower skin AGE levels. On average, their skin AGE levels were associated with people who were 2.07 years younger than themselves. Most iridoid consumers fell into three major daily intake groups. Differences in actual age and AGE associated age reveal a dose related trend. Those consuming 60 mg of iridoids per day had average AGE scores expected of those 0.46 years younger than themselves. The mean differences for the 120 mg and 240 mg groups were, respectively, 0.76 and 2.35 years less than noniridoid consumers. Among current nonsmokers, the difference between the reference group and iridoid consumers was somewhat larger than that observed for the total population. Among those who have never smoked, there was an even greater difference, with iridoid consumers having AGE levels that are associated with people 3.52 years younger than themselves. Linear regression analysis of the data obtained from this study further demonstrates an association between iridoid ingestion rates and lower AGE scores. The difference between participant age and AGE associated age is described by the following equation: −0.017  × mg iridoids ingested + 0.4538. The intercept of the regression model is 0.4538, but this is not statistically different than an intercept of 0 (*P* = 0.24). However, the slope of the trend, −0.017, is significant (*P* < 0.0001). This indicates that, for every mg of iridoids consumed in the diet, there is an expected decline in AGE levels, corresponding to 0.017 fewer years.

## 4. Discussion

The iridoids inhibited the nonenzymatic formation of fluorophores that results from the chemical interaction of reducing sugars and albumin. The* in vitro* results, when compared to aminoguanidine, may exclude one possible antiglycation mechanism of the iridoids. It is apparent that aminoguanidine is more active at lower concentrations. Even so, within the range tested, doubling of aminoguanidine concentration did not result in a doubling of activity. Aminoguanidine's anti-AGE activity involves its ability to act as a scavenger of highly reactive dicarbonyls that are Maillard reaction products [39]. Aminoguanidine reacts with glyoxal, methylglyoxal, and 3-deoxyglucosone to form triazine adducts. The reaction rate with glyoxal is first order, while it is more complex with methylglyoxal and 3-deoxyglucosone [40]. But all of these dicarbonyls are highly reactive, with the majority appearing to be scavenged at aminoguanidine concentration less than 1.25 mM, which is consistent with previous reports [41, 42]. The* in vitro* antiglycation activities of the iridoids, however, begin to increase dramatically above 2.45 mM. It is likely that these do not react with glyoxal in the same manner as aminoguanidine. Certainly, these iridoids do not form triazine adducts, as they contain no nitrogen. It is possible that* in vitro* reactions between dicarbonyls and the iridoids are not first order. Further, the* in vitro* anti-AGE action of iridoids may even involve different Maillard products. The exact mechanism of action still requires further investigation. But the data from the current study suggests that the mechanisms involved might not include first order reactions between the iridoids and dicarbonyls.

In the human pilot study, the average baseline skin autofluorescence value was higher than expected, based on average chronological age. This is not surprising, as inclusion criteria mandated that participants be overweight. Health status and lifestyle factors appear to influence AGE accumulation rates and, consequently, skin autofluorescence levels. For example, multiple reports describe elevated skin autofluorescence levels in diabetic patients [43–47]. Similarly, the presence of metabolic syndrome and adiposity is associated with increased skin autofluorescence [48, 49]. Oxidative stress is enhanced by central obesity [50–52]. This results in increased AGE accumulation and a concomitant elevation in skin autofluorescence [53].

An important consideration in interpreting the results of studies utilizing the AGE Reader is possible natural variation in skin autofluorescence. The reproducibility of various methods of skin autofluorescence measurement is reported to be fairly robust [54]. A previous reproducibility study with the AGE Reader revealed that repeated measurements over the course of a single day in both diabetic and nondiabetic individuals had a Bland-Altman percentage error of 5.03%. Further, the intraindividual seasonal variation among these people had a Bland-Altman percentage error of 5.87% [35]. Utilizing the same Bland-Altman method to compare baseline and 8-week skin autofluorescence results in our study, we found a percentage error of 41.86%. This is more than seven times greater than the expected daily and seasonal variations and further substantiates the conclusion that decline in mean autofluorescence is likely due to consumption of the iridoid enriched beverage.

The observations in this human pilot study are consistent with the results of clinical trials with noni juice, a dietary source of deacetylasperulosidic acid [32]. In heavy cigarette smokers, consumption of noni juice resulted in lowered plasma concentrations of superoxide anion radicals (SAR) and lipid hydroperoxides [26], as well as a reduction in lipid peroxidation-derived DNA adducts [55]. The probable role of deacetylasperulosidic acid in the antioxidant activity of noni juice was demonstrated by its ability to increase superoxide dismutase and catalase activity* in vivo* [56]. Loganic acid also reduced superoxide generation in human neutrophils activated by N-formyl-methionylleucyl-phenylalanine and arachidonic acid, in a concentration-dependent manner [57]. So aside from potential antiglycation activity, which involves inhibition of nonenzymatic chemical reactions, iridoids also appear to possess the ability to enhance the body's natural enzyme-based antioxidant defenses. This is important, as increased oxidative stress and AGE accumulation are closely tied together, with superoxide playing an important causative role [15, 58]. In fact, superoxide dismutase and catalase have long been thought to inhibit the formation of advanced glycation end products [59–61]. Therefore, control of superoxide concentration appears to be a major mechanism through which these two iridoids inhibit AGE formation.

The trends observed in the cross-sectional population study corroborate the results observed in the human pilot study. The iridoid associated decline in skin AGE levels among nonsmokers is more pronounced, with even greater declines among those who have never smoked. This is not surprising given the fact that smoking is a major accelerator of AGE formation. Tobacco smoke is an exogenous source of AGEs [62]. Further, cigarette smoke exposure increases systemic oxidative stress [63]. Tobacco smoke also upregulates the receptor for advanced glycation end products (RAGE), leading to inflammation and increased oxidant production, conditions which enhance AGE formation [17, 64]. AGE concentrations in the eye lens and coronary arteries of nondiabetic smokers are approximately 4-fold higher than in nonsmokers [65]. Skin autofluorescence has been consistently reported to be elevated among cigarette smokers [36, 37, 48, 66, 67] with smoking history having a significant effect on skin AGEs [68]. Also, there are apparent differences in AGE levels among current nonsmokers, depending on smoking history. For example, skin autofluorescence was lower among those who have never smoked than among ex-smokers [69]. Given the ability of cigarette smoke to increase oxidative stress and increase AGE production, it is expected that the anti-AGE activity of iridoids will be more easily detected among consumers who do not currently smoke. Accordingly, the decline in autofluorescence was less pronounced among former smokers than among those who have never smoked. A likely reason for this is that AGEs may persist in tissues for long periods of time. This is due to the cross-linking of glycated proteins with long half-lives, such as collagen, which makes them resistant to enzymatic degradation [70–73]. It is this AGE cross-linking of proteins that is thought to be responsible for “metabolic memory,” wherein some diabetic complications continue long after achieving glycemic control and where early intervention results in better long-term outcomes than later intervention [74–76]. Likewise, a longer history of smoking, as well as number of cigarettes smoked per day, will result in greater levels of persistent glycated protein cross-links. These persistent cross-links may mask some of the AGE reducing effect of iridoids, thereby resulting in less dramatic results among current smokers and ex-smokers. Even so, it does appear that iridoid intake is associated with reduced AGEs, as measured by skin autofluorescence.

## 5. Conclusion

*In vitro* testing of two dietary iridoids, deacetylasperulosidic acid and loganic acid, reveals that these possess potential antiglycation activity. Results of the human pilot study and the cross-sectional population study indicate that consuming dietary sources of these iridoids results in a reduction in AGE accumulation. Previous research also reveals that these iridoids may exhibit anti-AGE activity via induction of antioxidant enzyme activity, specifically superoxide dismutase and catalase. The extensive published evidence for accelerated aging in those with increased AGE accumulation and the effects of the iridoid enriched diet in our second substudy on tissue AGE accumulation suggest that consuming dietary sources of iridoids may be a helpful antiaging strategy.

## Figures and Tables

**Table 1:** Average differences between participant age and AGE associated age (ASA) among iridoid consumers and nonconsumers in a cross-sectional population study with 3,913 participants.

Daily iridoid intake	Average difference between age and ASA (± S.D.)
Reference group (*n* = 1123)	0.72 ± 15.93^*^
All iridoid consumers (*n* = 2790)	−2.07 ± 15.62^**^

Major subgroupings of iridoid intake	
60 mg (*n* = 487)	−0.46 ± 15.53^**^
120 mg (*n* = 1234)	−0.76 ± 14.91^**^
240 mg (*n* = 482)	−2.35 ± 16.28^**^

Nonsmokers	
Reference group (*n* = 922)	−0.76 ± 15.60^*^
All iridoid consumers (*n* = 2468)	−2.90 ± 15.25^**^

Never smoked	
Reference group (*n* = 728)	−0.51 ± 15.58^*^
All iridoid consumers (*n* = 1889)	−3.52 ± 15.32^#^

^*^The same as general population in previous published surveys (*P* > 0.05).

^**^ *P* < 0.001, compared to reference (0 mg iridoid) group.

^#^ *P* < 0.0001, compared to reference (0 mg iridoid) group and to general population survey data.
